# Electrochromic selective filtering of chronodisruptive visible wavelengths

**DOI:** 10.1371/journal.pone.0241900

**Published:** 2020-11-05

**Authors:** Maria Angeles Bonmati-Carrion, Javier Padilla, Raquel Arguelles-Prieto, Anna M. Österholm, John R. Reynolds, Juan Antonio Madrid, Maria Angeles Rol

**Affiliations:** 1 Chronobiology Laboratory, Department of Physiology, IMIB-Arrixaca, University of Murcia, Murcia, Spain; 2 Ciber Fragilidad y Envejecimiento Saludable, Madrid, Spain; 3 Department of Applied Physics and Naval Technology, ETSII, Technical University of Cartagena (UPCT), Cartagena, Spain; 4 School of Chemistry and Biochemistry, School of Materials Science and Engineering, Georgia Institute of Technology, Atlanta, Georgia, United States of America; University of Lübeck, GERMANY

## Abstract

We present evidence of pupil response modification, as well as differential theoretical melatonin suppression through selective and dynamic electrochromic filtering of visible light in the 400–500 nm range to minimize chronodisruptive nocturnal blue light exposure. A lower activation of intrinsically photosensitive retinal ganglion cells (ipRGCs), the first step for light to reach a human’s internal clock, is related to melatonin secretion therefore avoiding detrimental effects of excessive blue light exposure. Pupillary Light Reflex and Color Naming were experimentally assessed under light filtered by two different coloration states (transmissive and absorptive) of these novel dynamic filters, plus an uncoated test device, in 16 volunteers. Also, different commercial light sources at illuminances ranging from 1 to 1000 lux were differentially filtered and compared in terms of theoretical melatonin suppression. Representative parameters of the pupil responses reflected lower pupil constriction when the electrochromic filters (ECFs) were switched on (absorptive state, blue light is absorbed by the filter) compared to uncoated filters (control sample), but failed to do so under transmissive state (blue light passes through the filter) indicating less activation of ipRGCs under absorptive state (although no significant differences between states was found). Out of eight colors tested, just one showed significant differences in naming between both filter states. Thus, the ECF would have some protecting effect on ipRGC activation with very limited changes in color perception. While there are some limitations of the theoretical model used, the absorptive state yielded significantly lower theoretical melatonin suppression in all those light sources containing blue wavelengths across the illuminance range tested. This would open the way for further research on biological applications of electrochromic devices.

## Introduction

Our circadian system consists of a hierarchically organized network of structures responsible for endogenously generating ~24 h or daily rhythms that allow organisms to adjust their physiology and behavior, and to anticipate daily environmental changes. In mammals, the circadian system is composed of a central pacemaker located in the suprachiasmatic nuclei (SCN) of the hypothalamus, and peripheral oscillators in all organs and tissues. This system requires daily inputs to be reset and it presents different quantifiable outputs (reviewed in [1]). The main input to keep the circadian system entrained to the external time is the light/dark cycle [2], and a substantial difference between light levels (high) during the day and during the night (darkness) has been related with a better circadian synchronization and status [3].

However, this natural light/dark cycle is systematically disrupted in our daily routine, particularly in urban environments (reviewed in [1]). This is especially relevant for shift workers, frequently under reduced or even inverted light/dark cycles, which often leads to chronodisruption and suppression of melatonin [2], a hormone with multiple physiological functions including resetting of the daily the central pacemaker [4,5]. Chronodisruption is defined as a significant disturbance of the internal temporal order of physiological, biochemical, and behavioral circadian rhythms, as well as a disruption of the link between internal and external times [6]. When prolonged or repeated, this disruption has been associated with higher prevalence of various health impairments [7,8], such as metabolic syndrome [9,10], cardiovascular diseases [11], cognitive impairments [12], and a higher incidence of breast cancer [13,14], to name a few.

To synchronize our circadian system, light enters the SCN through the retina. Light inputs are codified by different types of photoreceptors: S-cones, M-cones and L-cones, with maximum sensitivity around 420, 530, and 559 nm [15], respectively, are in charge of image forming in daylight conditions, while rods, centered at 498 nm [16], mainly function in low light conditions in terms of vision (although they can contribute to circadian entrainment under high light intensities [17]). A third class of photoreceptors, which constitute the first step for light to enter the circadian system, are the intrinsically photosensitive retinal ganglion cells (ipRGCs), a specific type of ganglion cell containing melanopsin. These cells are directly excited by light with a peak of sensitivity demonstrated around 480 nm [18], although they can also be activated by other wavelengths [19]. These ipRGCs project to the SCN and other non-image forming brain areas, such as the olivary pretectal nucleus, a control center for the pupillary light reflex (PLR) [20–24]. Thus, ipRGCs participate in a common pathway for the PLR and circadian entrainment. Not surprisingly, the most efficient wavelengths (λ_max_ = 470–525 nm) for entraining the circadian system and inhibiting melatonin secretion in humans [25–27] are close to the wavelength range where the ipRGCs exhibit their maximum sensitivity. As a result, not all light wavelengths are equally chronodisruptive throughout the day. Blue light (460–480 nm [28]) is beneficial during the day and has been reported to have positive effects on well-being, cognitive performance, and circadian status [29]. However, it has been shown to be disruptive at night, inducing the strongest melatonin suppression at around 460 nm [26,30].

Consequently, a possible solution for incorrect light exposure is through selective and dynamic suppression of blue light, with transmission during the day and filtering at night, which can be done either by adaptive lighting systems or by using dynamic filters. Electrochromic materials have the ability to gradually and reversibly modify their absorbance/transmittance spectrum in specific wavelength ranges in response to a small voltage and are therefore ideal candidates to be incorporated into user-controlled, dynamic filters. Conjugated polymers are particularly interesting for this application as their absorption spectrum can be easily modified synthetically to absorb the wavelength of interest [31,32].

The aim of this study was to evaluate ipRGC activation through pupillometry, as well as color perception (through a color naming test) and theoretical retinal photoreceptors activation and melatonin suppression, using a dynamic electrochromic filter (ECF) incorporating the electrochromic polymer poly[bis(ethylhexyloxy)thiophene-dimethoxyphenylene], referred to here as ECP-Yellow. ECP-Yellow absorbs light in the 400–500 nm range and therefore appears yellow to the eye when in its blue light blocking state with an absorption maximum at 446 nm. Upon application of a small voltage in an electrochemical device, this polymer can switch to a color neutral and fairly transmissive state throughout the visible range. This ability to dynamically filter light is used to modify the pupil responses after light stimulus, in order to prevent specific photoreceptor activation that is linked to circadian activation. Our hypothesis was that PLR parameters related with ipRGC response would reflect greater pupil constriction when the filter was in the transmissive state than in the absorptive state when using the same photon flux at corneal level. It could be expected that color perception (in our case, color naming hit rate) would be compromised under the absorptive state for colors with high blue content while those colors with greater red-green content could be more likely misnamed under transmissive state. Similar perception under both states is beneficial and even critical for safety if they are to be used in work environments where the ability to discriminate between colors is important. Also, we expect light sources filtered under absorptive state to produce lower theoretical melatonin suppression. The results obtained in this study open the way to explore the possibility of dynamically influence the light-induced biological signals related to our internal clock, by means of selectively blocking targeted wavelength ranges through electrochromic filters.

## Materials and methods

### Participants

The participants were 16 healthy non-smoking volunteers (8 women) between 19 and 50 years of age (36.25 ± 8.12 years, mean ± SD). None of them reported any medical or mental disorders or to be taking any medication that could affect circadian rhythms. None of the participants were shift workers and no one had crossed more than two time zones in the two months prior to study admission. None of the volunteers exhibited color blindness according to Ishihara test. Volunteers received appropriate information about the study protocol, signed a written informed consent form (see SI, Annex 1) in accordance with Helsinki Declaration of 1975, as revised in 2008, before being enrolled into the study and were compensated for their participation. This research project was approved by the University of Murcia Ethics Committee (ID 2000/2018) and all research was performed in accordance with relevant guidelines/regulations.

### Dynamic filters

The dynamic filters used were electrochemical devices composed of ECP-Yellow as active color changing material and vanadium(V)oxide (V_2_O_5_) as transparent non-color changing complementary material. ECP-Yellow (synthesis procedure described in S1 File) shows a color transition between a transparent oxidized state and a vibrant yellow neutral state, where both states can be gradually and reversibly obtained electrochemically. Transmission properties, together with the electrochemical behavior of both materials, are shown in S1 Fig in S1 File. The static filtering properties of ECP-Yellow (as single films in their neutral state) in the 350–500 nm range and under different illumination sources, were confirmed (S2 Fig in S1 File).

Prior to the construction of the devices used in this study, an optimization procedure was carried out for ECP-Yellow, in order to maximize the resulting contrast (i.e. the difference in transmittance between transmissive and absorptive states for the wavelengths of interest). By individually characterizing the optical and electrochemical response as a function of increasing thickness films, we were able to identify the properties and the deposition conditions to obtain the maximum electrochromic contrast (S3 Fig in S1 File).

Electrochromic filters were fabricated with a “sandwich-type” architecture (S4a and S4b Fig in S1 File) ECP-Yellow and V_2_O_5_ films were spray coated over indium tin oxide (ITO) substrates via an automated CNC routine. Devices were assembled using a photo-crosslinkable electrolyte gel. More details on the deposition, film optimization and device construction can be found in the SI.

The resulting devices were optically and electrochemically characterized, obtaining a reproducible contrast of 32 ± 3% at 446 nm for all devices, when applying 1.7 V and -0.2 V (to obtain transmissive and absorptive states, respectively). Transmittance spectra of both states, together with that corresponding to a control device (device solely composed of the transparent substrates and electrolyte, without ECP-Yellow or V_2_O_5_) are shown in S4c Fig in S1 File.

### Pupil recording

**Fig 1:**
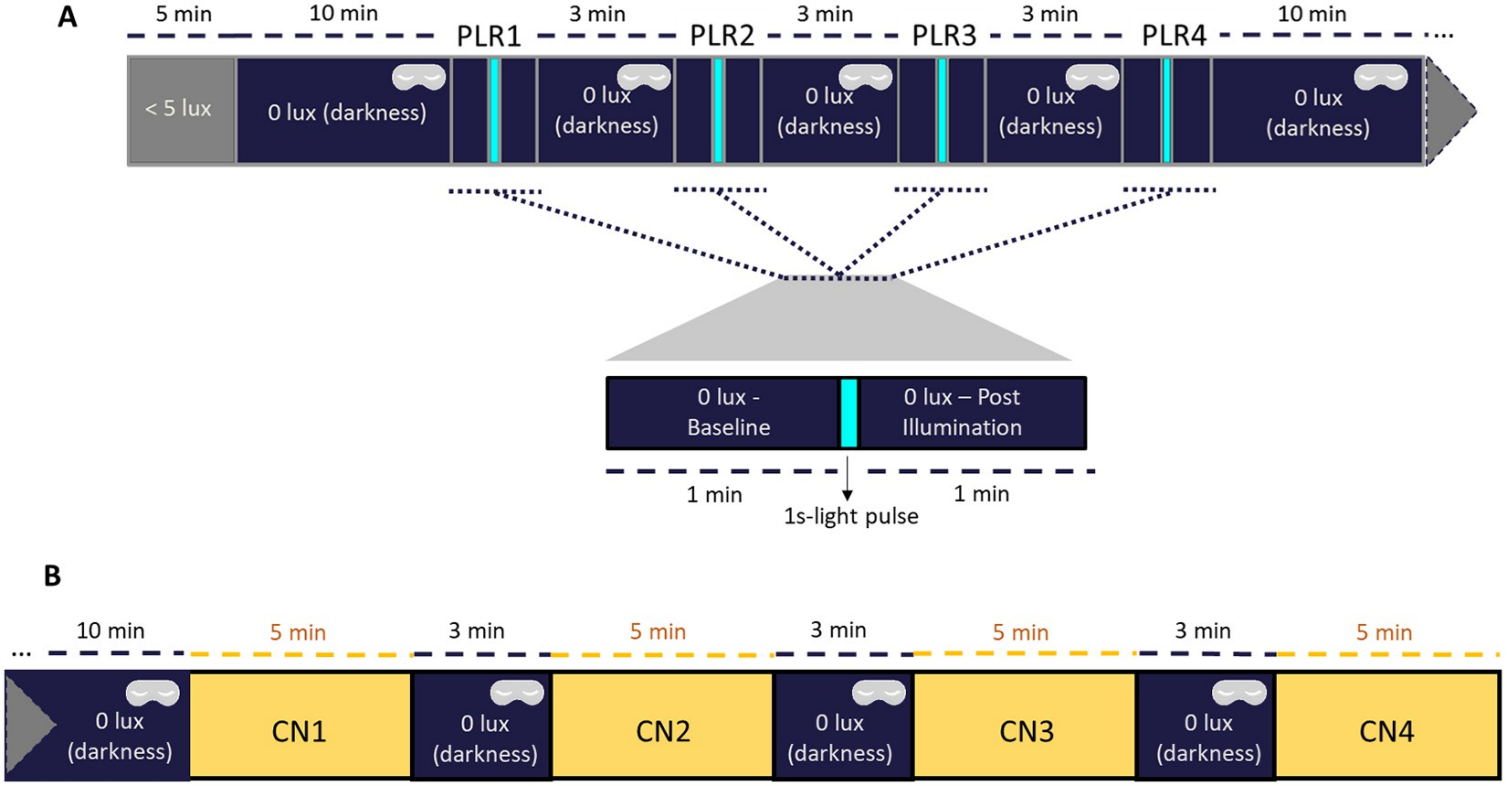
A) Experimental design for pupil light reflex (PLR) assessment and B) color naming (CN) tests. (A) Each pupil recording (PLR) consisted of 1 minute of baseline pupil size in darkness (dark blue areas without eye mask), followed by 1 second light stimulus (turquoise areas) and 1 minute of recovery in darkness (dark blue areas without eye mask). Between each PLR, the participant remained in darkness for 3 minutes, also wearing an eye mask (dark blue areas with an eye mask). (B) Each CN test lasted 5 minutes. Between each CN test, the participant remained 3 minutes in darkness wearing an eye mask (dark blue areas with an eye mask).

A randomized, within-subject study was conducted. Full details of the study protocol are presented in Fig 1. All the tests were performed at similar time of day to limit any circadian influence in ipRGC contributions to PLR [33,34] and they were performed according to the published standards in pupillography [35]. The participants arrived at the laboratory and stayed seated in dim light (< 5 lux) for 5 minutes in order to let their eyes progressively adapt to low light intensity. After that, and prior to the first PLR test (Fig 1A), the participant remained in darkness (0 lux + eye mask) for 10 minutes to avoid any light exposure prior to the first tested light stimulus (filtered by first filter state). Pupil recordings (220 frames/second; ViewPoint Eye Tracker^®^, Arrington Research Inc., Scottsdale, AZ) were performed on the left eye (infrared illuminated), starting with 1 minute in darkness for the assessment of baseline pupil size. Then, a 1-second light pulse (filtered through one filter state per recording) was applied onto the right eye while left eye continued being recorded (consensual PLR). After the light pulse, the left pupil was tracked for 1 minute to document the pupil size recovery after the light stimulus. Following this, the participant remained in total darkness wearing an eye mask for 3 minutes, while the state of the filter was changed by applying short voltage pulses (10 s) of 1.7 V to achieve the transmissive state or -0.2 V for the absorptive state. These steps were repeated for each filter state tested (1 light stimulus per filter state) in randomized order: transmissive, absorptive, control sample, or no filter.

The pupil diameter was pre-processed using Pupilabware^®^ software [36], including filtering, determination of baseline (mean pupil diameter during the 60 s in darkness prior to the light stimulus) and pupil size normalization, i.e. ratio of the measured pupil diameter divided by the baseline pupil size.

Regarding the pupil outcome parameters, relative maximum constriction (mostly mediated by rods and melanopsin) with respect to the basal size was obtained by subtracting the percentage of the minimum pupil size reached from 100 (the baseline pupil size); time to minimum as time required to achieve the relative maximum pupil constriction, and velocity of pupil constriction as $\left(\frac{maximum\;constriction}{time\;to\;minimum\;diameter}\right)$. Post-illumination pupil responses were measured 6 seconds (6s-PIPR, mediated by melanopsin [37,38]) after the light pulse, averaging pupil constriction between 5.5 and 6.5 seconds after light pulse. In order to integrate both transient (early) and sustained response (late), the area under the curve from 1^st^ to 6^th^ second (AUC_1-6_) was calculated as $AUC=(AUC\sum_{t0}^{t1}100-NPS)$, where t_0_ is the initial time point of pupil response (1 s) and t_1_ is the end time (6 s), 100 is the baseline pupil size, and NPS is the normalized pupil size. Similarly, to represent melanopsin and rod function, AUC_1-1.7_ was calculated from 1 to 1.7 s after light onset.

**Fig 2:**
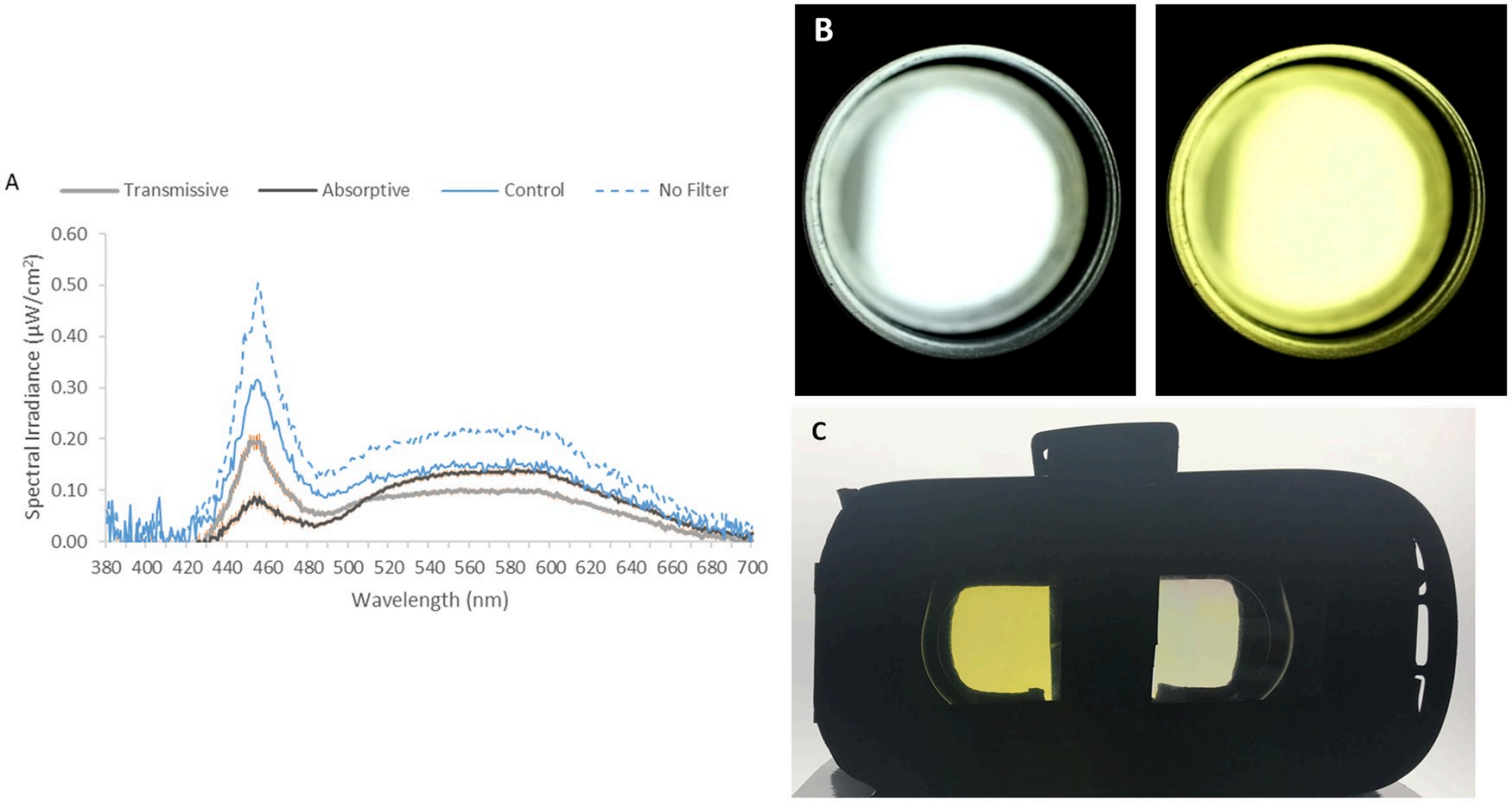
A) Averaged light spectra for the unfiltered light source (blue dashed line, no filter), light through uncoated filter (control sample, blue line), under transmissive (grey line) and absorptive (black line) states. For unfiltered light and the uncoated control filter, just one measurement was performed, while for ECFs, 11 filters were measured under both states. Orange vertical lines for transmissive and absorptive states indicate standard error of the mean (SEM). B) Images of pulse light as the user perceived them with the filter in transmissive and absorptive states. Estimated RGB values: transmissive (188,192,188), colored (219, 210, 102). C) Filters binocularly implemented in a commercial virtual reality case that participants wore for color naming tests. In the photograph, left filter is in the transmissive state and right filter is in the absorptive state. During actual experiments, both ECFs were in the same state.

A polychromatic commercial light source (LED panel, 5300–5700K, Osram, NTSistemas, Murcia, Spain, 59x59 cm, spectrum shown in Fig 2A (blue dashed line)) was used to be filtered through each filter’s state. Spectra (Fig 2) were measured for each filter and state at eye level by a calibrated spectrometer (Ocean Optics BV, Dunedin, Florida, USA). Light stimuli were applied through a cylinder (101 cm long, 20 cm diameter) with the dynamic filter located just in front of the eye, inserted in an eye piece (4 cm diameter) (Fig 2B), so all light coming into the eye was filtered.

### Color naming tests

After four PLR recordings, the participant remained in total darkness for 10 minutes. Then, four color naming tests (CN) (Fig 1B) (one test/filter state) were performed over the course of ~30 minutes. The same light source previously described for PLR assessment was used, in this case located above the participant, in vertical projection. Filters were binocularly implemented in a commercial virtual reality case that each participant wore (Fig 2C). The participants were individually shown eight different Munsell (DG Colour, Worldwide IT Ltd, Warminster, UK) color cards (refs. 7.5R 6/4, 5Y 6/4, 5GY 6/8, 2.5G 6/6, 10BG 6/4, 5PB 6/8, 2.5P 6/8, 10P 6/8, for details see S1 Table in S1 File), and they were asked to self-assign (with no restrictions) a color name to each card. The reason why these colors were chosen was that they cover a wide range in blue/red-green content (see S1 Table and S5 Fig in S1 File). Then, eight of those cards (repetitions possible) were presented to the volunteer during 30 seconds each in a random order, with a visual angle of 29.4166° (29° 24’ 1.00'')—38.5801° (38° 34’ 0.81''). The volunteer was then asked to identify each color according to the names previously self-assigned. The number of correct responses was assessed for each participant under each filter condition (control filter, absorptive, and transmissive state), eight being the maximum correct responses and zero the minimum. Between each filter condition, the participant remained in total darkness for 3 minutes. For the analysis of each color hit rate under each condition, each hit number was divided by the times that card was shown to each participant under each filter state.

### Photoreceptor activation

The photoreceptor activation was assessed as α-opic log photon irradiance (log quanta/cm^2^/s) for each dynamic filter states, using the Equivalent Daylight (D65) Illuminance Toolbox beta version E1.05 developed by the International Commission of Illumination (CIE) [39]. Each parameter (rhodopic, cyanopic, melanopic, chloropic, and erythropic) represents the excitation of each type of photoreceptors (rods, S-cones, ipRGC, M- and L-cones, respectively), based on each photoreceptor’s estimated sensitivity curves [39]. All filters were spectrally analyzed in their transmissive and absorptive states, including the fixed values for the uncoated control filter (spectrally measured just once as it can be assumed to be unchanged throughout the experiment).

### Theoretical melatonin suppression: ECF application to commercial light sources

In order to theoretically assess the effects of the filters on melatonin secretion we applied the experimentally obtained device transmittance spectra to different commercial light sources (5700 K LED, 3000 K LED, RGB LED, RGV LED, fluorescent, incandescent and mercury vapor sources, amber LED and sun light, see S6 Fig in S1 File) at different light intensities (from 1–1000 lux). Then, the circadian model proposed by Rea et al. [40] was applied to the resultant light spectra and intensities. This model accounts for participation of ipRGCs as well as rods and cones in circadian phototransduction via neural connections, including spectra opponency, in the outer plexiform layer of retina. Then, the CS (Circadian Stimulus) index was examined, which is equivalent to melatonin suppression in %.

### Statistical analysis

Repeated measures ANOVA for PLR and CN tests were performed, and significant results compared *post-hoc* by Bonferroni test. For physical characteristics of light and theoretical photoreceptor activation, 1-sample and paired T-Tests were used (the uncoated control filter was spectrally measured only once considering that it remains unchanged, therefore providing a fixed value for each parameter). Normality of the data was checked using Shapiro Wilk’s test. The significance level was set in both cases at p < 0.05. All results are expressed as mean ± standard error of the mean (SEM).

## Results

### Physical characteristics of filtered light

**Fig 3:**
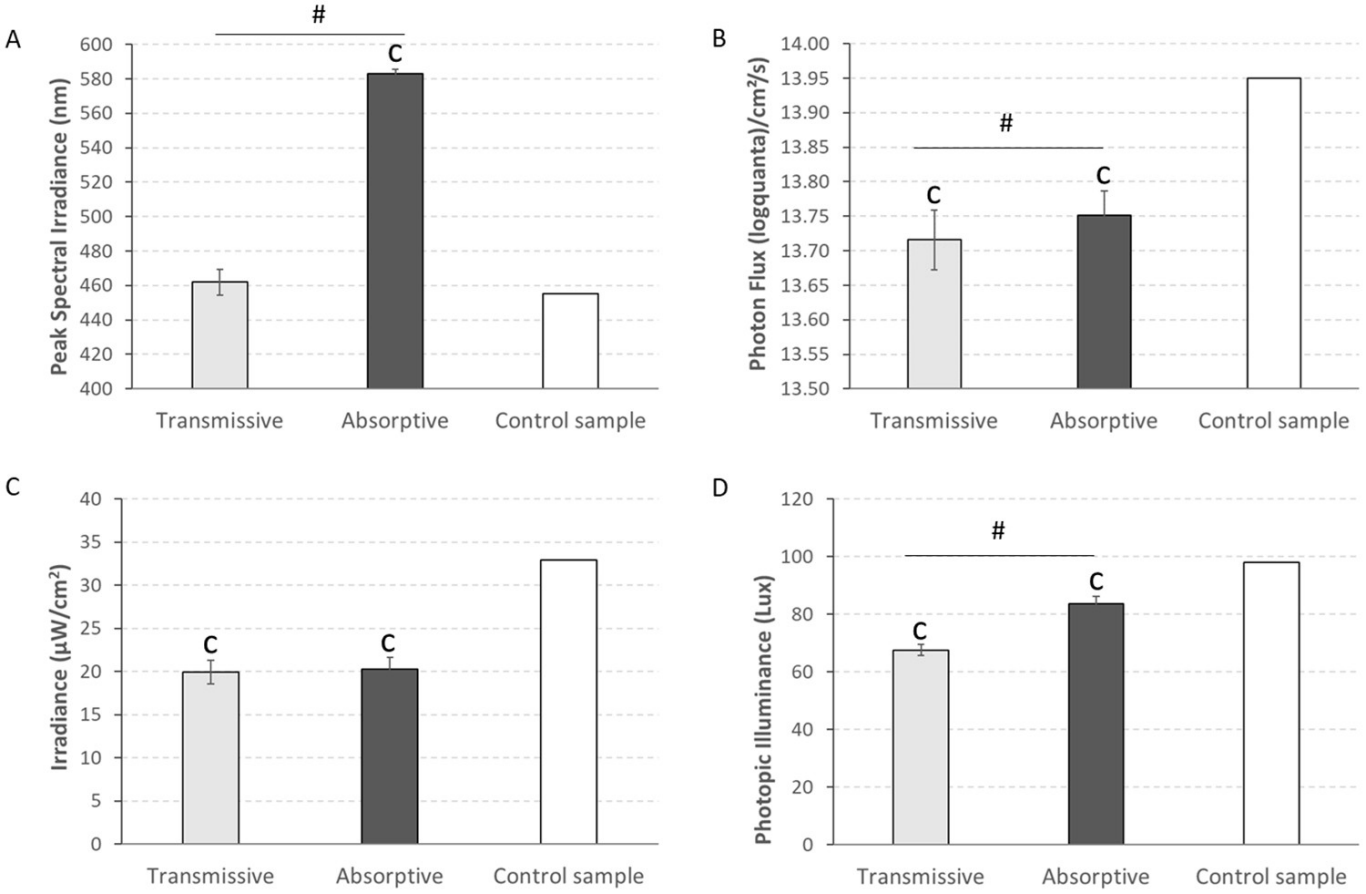
A) Peak of spectral irradiance, B) photon flux, C) irradiance and D) photopic illuminance of the light under transmissive (N = 11) and absorptive (N = 11) conditions as well as that for a control sample (N = 1). Vertical bars indicate SEM.1-sample T-Test was used to compare both transmissive and absorptive states with the control sample and paired T-Test was performed to compare between both states. ‘c’ indicates significant differences with the control sample (1-sample T-Test, p < 0.001) while ^#^ indicates significant differences between transmissive and absorptive states (paired T-Test, p < 0.001).

In order to physically characterize each light stimulus, we spectrally measured the light stimulus transmitted through each lens and state. As shown in Fig 3A, the ECF in the absorptive state peaked at significantly longer wavelengths (583 ± 2 nm) when compared, not only to the control filter spectrum (455 nm) (1-sample T-Test, p < 0.001), but also to the ECF in the transmissive state (462 ± 8 nm) (Paired T-Test, p < 0.001). Photon flux (Fig 3B), irradiance (Fig 3C), and photopic illuminance (Fig 3D) for both ECF states were significantly lower than for the uncoated filter (1-sample T-Test, p < 0.001). When comparing the absorptive and transmissive states, the irradiance was not significantly different between the two (Paired T-Test, p > 0.05), but the photopic illuminance was significantly lower for transmissive (67.53 ± 1.93 lux) than the absorptive (83.67 ± 2.45 lux) state. The same trend was observed for the photon flux, although in this case, the differences were much smaller (13.72 ± 0.04 log quanta/cm^2^/s for transmissive and 13.75 ± 0.03 log quanta/cm^2^/s for absorptive, Paired T-Test, p < 0.001, which allows us to discard influence of light intensity on PLR.

The transmittance spectrum of each filter state is shown in S4c Fig in S1 File. It can be observed that transmittance values were lower for the absorptive ECF state in the desired wavelength filtering range, between 400–520 nm. Interestingly, those filtering effects are inverted for in the 520–700 nm range (transition defined by the isosbestic point at ~520 nm). Calculation of an integrated transmittance over the visible range reveals that similar values were obtained for both states (52 ± 4% for transmissive *vs*. 53 ± 3% for absorptive).

### Pupil light reflex

**Fig 4:**
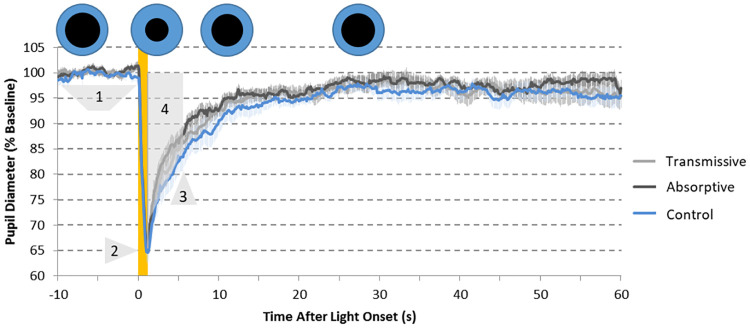
Pupillary light reflex (PLR) recordings (N = 16) under 1-s light stimulus filtered by the control sample (blue line, used as control), the ECF in a transmissive state (grey line) and the ECF in an absorptive state (black line). The yellow area indicates the 1-second light stimuli. Pupil diameter is expressed as percentage of each recording baseline. Pale lines represent SEM. Parameters assessed for PLR. 1: Baseline (~100%); 2: Maximum constriction (% with respect to the baseline); 3: 6-s PIPR (% constriction 6 seconds after light stimulus); 4: area under the curve from 1 to 6 s after light onset (AUC1−6, yellow), expressed in A.U. Blue (iris) and black (pupil) circles qualitatively represent the evolution of the pupil size (black circles) during a standard PLR test.

**Fig 5:**
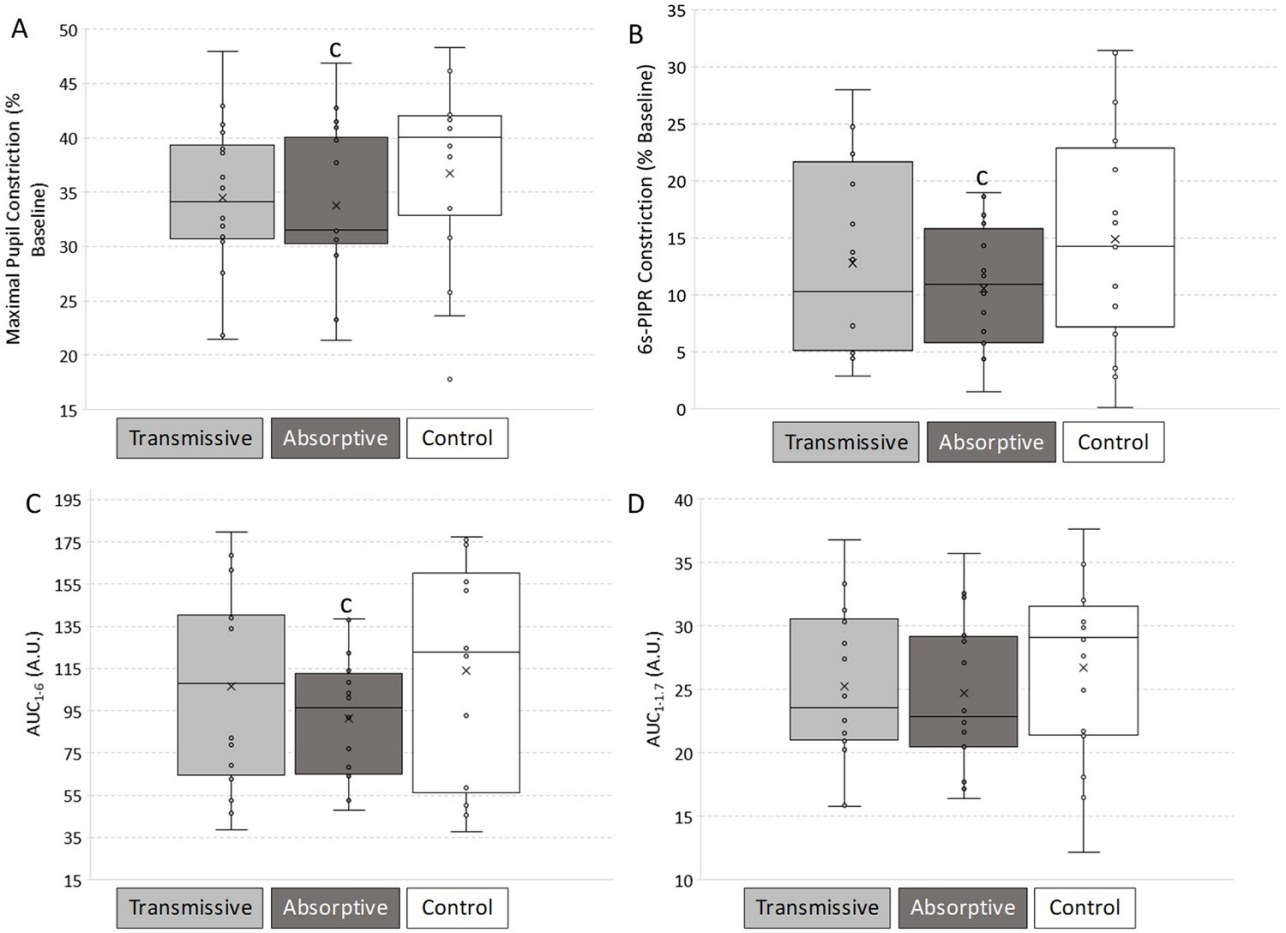
Biological validation of filtered lights (N = 16). Pupillary Light Reflex (PLR) parameters calculated under light filtered with uncoated (used as control), transmissive and absorptive states: A) Maximum pupil constriction, with respect to the baseline; B) post-illumination pupil response 6 seconds after the end of the 1-s light stimulus (6s-PIPR), expressed as % constriction respect to baseline; C) Area Under the Curve from 1 to 6 seconds from the beginning of the light stimulus (AUC_1-6_) and D) Area Under the Curve from 1 to 1.7 seconds from the beginning of the light stimulus (AUC_1-1.7_) both calculated from baseline (100%). Repeated measures ANOVA with Bonferroni *post-hoc* was performed. ‘c’ indicates significant differences with uncoated control filter (p < 0.05).

In our study, the main biological test is the PLR, which followed the expected dynamics under all tested light conditions (Fig 4). This included the rapid and transient constriction and a more sustained partial pupil constriction, which lasted several seconds (up to 60) after stimulus termination. The maximum pupil constriction (Fig 5A) reached 36.69 ± 2.12% for the PLR measured under the control sample, while the maximum constriction was 34.48 ± 1.82 and 33.73 ± 1.89% for transmissive and absorptive states, respectively (repeated measures ANOVA, F = 5.415, df = 2, p = 0.010). Only the absorptive state of the ECF was significantly different from the uncoated control filter (Bonferroni *post-hoc*, p = 0.042), which indicates lower activation of the photoreceptors involved in this part of the PLR under absorptive state, and not under transmissive.

The post-illumination pupil response assessed 6 seconds after light stimulus (6s-PIPR) was also affected by the state of the ECF (repeated measures ANOVA, F = 4.195, df = 2, p = 0.025). 6s-PIPR for the transmissive state (12.79 ± 2.10%) did not differ from control sample and tended to be greater than for the absorptive ECF (Bonferroni *post-hoc*, p > 0.356). Indeed, the lowest constriction at this time point was displayed with the absorptive ECF (10.54 ± 1.37%), which was significantly lower than for the control sample (14.87 ± 2.46%) (Bonferroni *post-hoc*, p = 0.042) (Fig 5B).

A more integrative PLR parameter is the area under the curve from 1 to 6 seconds after light pulse onset (AUC_1-6_) (Fig 5C) as this provides integrated information on both transient and post-illumination pupil responses, with rhodopsin and melanopsin contributions. This value was also affected by the filter state (repeated measures ANOVA, F = 7.070, df = 2, p = 0.004). Values found for the absorptive ECF (91.47 ± 8.38 AU) were significantly lower than those found for the control sample (113.84 ± 15.79 AU) (Bonferroni *post-hoc*, p = 0.027) while the corresponding values for the transmissive ECF (106.78 ± 13.29 AU) were not. In order to isolate melanopsin and rod function, we also calculated AUC from 1 and 1.7 after light onset (AUC_1-1.7_) (Fig 5D). In this case, however, values under absorptive state (24.67 ± 1.50) only showed marginal significance (Bonferroni *post-hoc*, p = 0.062) when compared to the control sample (26.73 ± 1.76). No significant differences were shown between both states either.

### Color naming

In order to evaluate the ability of these filters to keep the color perception constant, a color naming test was performed. Taking global results, we did not find any significant differences for the filter states (repeated measures ANOVA, F = 2.005, df = 2, p = 0.154), although a tendency for higher number of coincident color names was found for the control sample (6.75 ± 0.37) compared to transmissive (6.25 ± 0.28) or absorptive (5.81 ± 0.40) ECF. When hit rate was individually analyzed for each color, as expected, those with higher blue content (>33%, S1 Table and S5 Fig in S1 File) tended, in 75% of the tested colors (#1, #6, #8), to show greater hit rate under transmissive than under absorptive state (S7 Fig in S1 File), while colors with higher red/green content (>67%, S1 Table and S5 Fig in S1 File), also in 75% (#3, #4, #5) of the tested colors, tended to show better hit rate under absorptive than transmissive state (S7 Fig in S1 File). These expected tendencies did not show significant differences between states (p > 0.322), except for color #2, which showed better naming hit rate under transmissive than under absorptive (repeated measures ANOVA, F = 7.604, df = 1.427, p = 0.007) despite having low blue content.

### Theoretical photoreceptor activation

**Fig 6:**
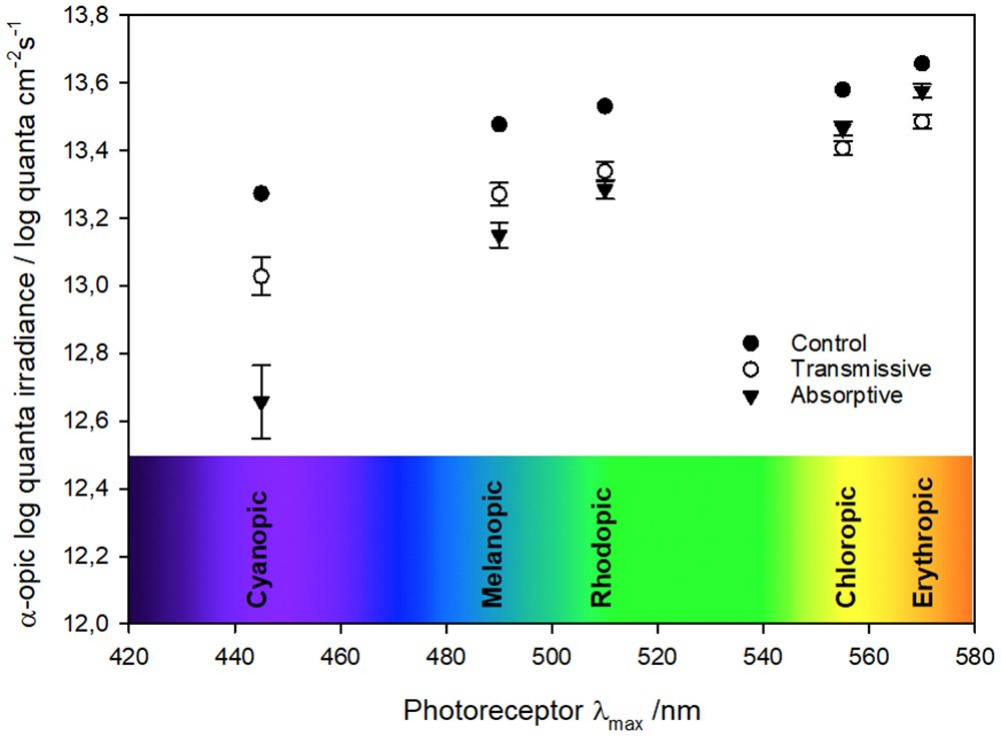
Theoretical photoreceptor activation for the studied devices in the three filtering states (uncoated, transmissive and absorptive) as a function of each photoreceptor wavelength maximum sensitivity (λ_max_). Averaged theoretical photoreceptor activation (CIE α-opic log photon irradiance, log quanta/cm^2^/s) under control (N = 1), absorptive (N = 11) and transmissive (N = 11) filter states: rods (rhodopic, λ_max_ = 510 nm), S-cones (cyanopic, λ_max_ = 445 nm), melanopsin retinal ganglion cells (melanopic, λ_max_ = 490 nm), M-cones (chloropic, λ_max_ = 555 nm) and L-cones (erythropic, λ_max_ = 570 nm).

To gain further insight into contribution of the different wavelength ranges to the pupil response, theoretical photoreceptor activation (Fig 6) was calculated in all filters according to CIE Light Toolbox [39]. When compared to the control sample, all photoreceptors were less activated when light was filtered through the ECF filter, regardless of the state of the filter (1-Sample T-Test, p < 0.003). When comparing the transmissive state to the absorptive state, rods (rhodopic), short-wavelength (S-) cones (cyanopic), and melanopsin RGCs (melanopic) were more activated when the ECF was in the transmissive state (13.34 ± 0.03, 13.03 ± 0.06 and 13.27 ± 0.03 log quanta/cm^2^/s, respectively) than in the absorptive state (13.29 ± 0.03, 12.66 ± 0.11 and 13.15 ± 0.04 log quanta/cm^2^/s, respectively). However, medium (M-) (chloropic) and long (L-) (erythropic) wavelength cones were more activated under the absorptive (13.46 ± 0.02 and 13.58 ± 0.02 log quanta/cm^2^/s, respectively) than the transmissive state (13.41 ± 0.02 and 13.49 ± 0.02 log quanta/cm^2^/s, respectively) (Paired T-Test, p < 0.004).

### Theoretical melatonin suppression: ECF application to commercial light sources

**Fig 7:**
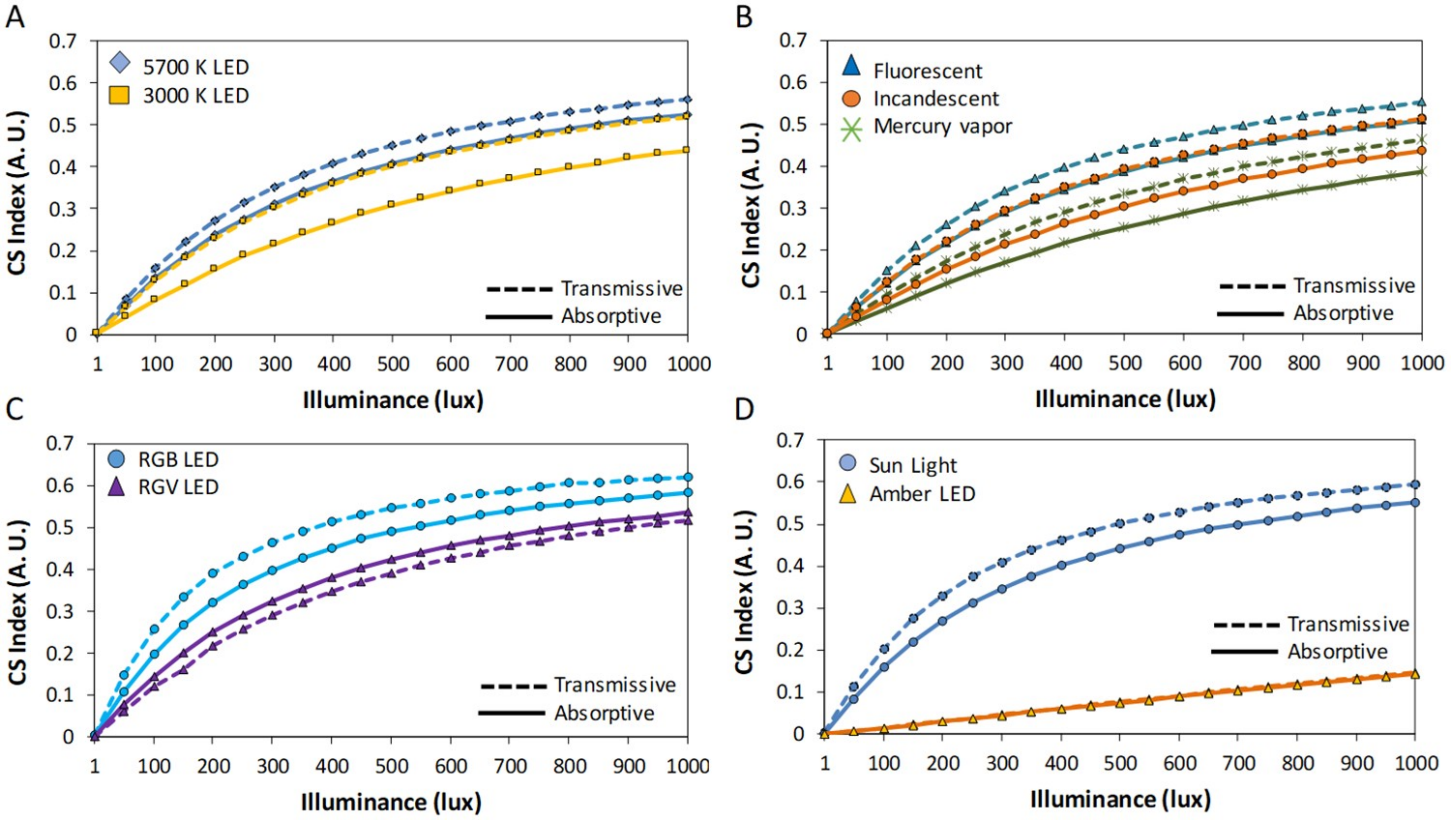
Theoretical melatonin suppression (according to Rea et al. 2010 model) for absorptive (solid lines) and transmissive (dashed lines) states filtering light spectra for: (A) 5700 (blue diamonds) and 3000 K (orange squares) LEDs, (B) fluorescent (blue triangles), incandescent (orange circles) and mercury vapor (green crosses) light sources, (C) RGB (blue circles) and RGV (purple triangles) LEDs, and (C) Sun light (blue circle) and amber LED (orange triangle). CS Index is equivalent to melatonin suppression (in %, thus a 0.5 indicates a 50% suppression).

In order to put our results into context, we performed simulations for melatonin suppression under different commercial light sources filtered by transmissive and absorptive states (Fig 7) at 1–1000 lux. In general, absorptive state reduced theoretical melatonin suppression up to 10% (CS index, equivalent to melatonin suppression in %) under all light sources containing blue wavelengths (all except RGV and amber LEDs). Surprisingly, filtered 5700 K LED under absorptive state became as melatonin suppressive as 3000 K LED filtered by transmissive state (Fig 7A), while filtering the latter with absorptive state significantly reduced its potential melatonin suppressive action. Similarly, fluorescent light source filtered by absorptive state reduced its theoretical melatonin suppression to reach incandescent values when filtered by transmissive state. Thus, fluorescent, incandescent, mercury vapor (Fig 7B), RGB (Fig 7C) and Sun light (Fig 7D) reduced theoretical melatonin suppression under absorptive state. Regarding illuminance levels, the differences between light sources and filter states became greatest from 100 to 1000 lux, being smaller at low intensities.

## Discussion

This study, conceived as a proof of principle, demonstrates a novel application of dynamic blue light filtering by electrochromic materials to a physiological process such as pupil light reflex, anatomically related to circadian entrainment. One of the main concerns when filtering light is the potential change in color perception, especially in work environments where this involves safety issues, such as in healthcare-related facilities. Although color is not considered as an intrinsic object property [41], it is true that color identification is habitually made under standard commercial light sources, so it is important that color perception does not significantly differ with the different filter states. In our study, we used a very simplified system to check whether color identification remained being possible with this novel dynamic filtering system. Although color perception can be assessed by alternative methods, the advantages of the color naming test used in this study are its simplicity and the short time it requires to be performed. In addition, the participants self-assigned the original color name with no restrictions, so this test can provide a reliable picture related to the stability of color perception under this filtering system. Indeed, although we found a slight tendency for a slightly lower number of coincident color names under the absorptive ECF state, these filters could be promising candidates to be used in contexts where color is relevant. Although only in one color the differences were in the opposite direction, our results, as expected, showed some tendencies (non-significant) depending on each color’s RGB content. Thus, colors with greater content in blue tended to be named incorrectly slightly more frequently under the absorptive than under the transmissive state, while the opposite tended to happen with those colors with higher red/green content. The potential usefulness of this filtering system to reduce melatonin suppression was tested by means of mathematical modelling [40]. This model, with the limitations discussed below, showed best theoretical melatonin protection under absorptive state within the range 100–1000 lux in blue-containing light sources.

Considering its physiologic relationship with circadian entrainment, we considered PLR as the main outcome to be assessed in this study. We found a pupil dynamic similar to that previously described [37,42–44] in all pupil recordings performed and with all the tested filters’ states (transmissive, absorptive and uncoated).

The post-illumination pupil response assessed 6 seconds after light stimulus (6s-PIPR) is the most accepted parameter for ipRGC activation [37]. Our results indicate that using these dynamic filters in transmissive and absorptive states during day and night, respectively, may protect ipRGCs from activation by light at night, while permitting it during the day, as under natural light conditions. However, more functional and physiological studies will be needed to confirm the potential of this filter to protect circadian clock from disruption by light at night. The AUC_1-6_ followed the same tendency, although in this case, this parameter involves both rhodopsin and melanopsin effects, both contributing to the early redilation phase. Moreover, under this wide-band calculation (5 seconds), the contribution of cones could not be ruled out either.

With the exception of AUC_1-1.7_ (marginally significant differences), all PLR parameters reflected less constriction under the absorptive state with respect to control sample (similar to that previously found for static yellow filters, reviewed in [45]), while the transmissive state did not produce significant changes in PLR compared to the control. However, when comparing both states (transmissive and absorptive) to each other, we did not find any significant differences in any PLR parameters. There are several possible reasons for this limitation. First, the irradiance achieved is low under both states, which could be the reason for the small effect size. Second, there are some intrinsic optical limitations of the studied devices in their ability to modulate transmission. Although the electrochromic polymer used in this study demonstrates an optical contrast up to 59% at 446 nm [46], the necessary inclusion of additional layers, especially that coming from ITO substrates, lowers the overall contrast of device to ca. 30% decreasing the net optical modulation. Third, the intrinsic characteristics of the device spectra may play a role. The pupil responds to the entire wavelength range, not just the wavelength range that is modulated by the ECF. Compared to the ECF in the transmissive state, the transmittance values were lower for the absorptive ECF state between 400–500 nm, but higher in the 500–700 nm range, therefore inverting the filtering effects. The calculation of the integrated transmittance over the visible range revealed that similar values were obtained for both, which is a possible explanation for the negligible difference in PLR parameters between the two states.

Finally, there is also the complexity of the retina’s response to polychromatic light. In this case, there is additional complexity as the wavelengths that are enhanced depend on the state of the polymer. Specifically, longer wavelengths are enhanced when the ECF is in the absorptive state polymer and shorter when it is in its transmissive state. The retina pathways involved in the ipRGC activation are complex, and include intrinsic (direct) and extrinsic (indirect, through rods and cones) pathways [25,26]. Also, the described spectral opponency between S-cones and both L- cones and ipRGCs [47–49] would explain why differences between the two states were not statistically significant, even though there was a clear tendency for the absorptive state to produce lower pupil constriction.

Regarding the light intensity, even considering a 0.3 correction for optical media, the photon fluxes used in this study were above the photopic threshold [50]. Although in the lower range, which could explain the low PIPR values, they are within the limits for melanopsin activation [51]. One strength, regarding its methodological validity, is that photon flux and irradiance obtained under both states were very similar, and as a result, a possible influence of light intensity on PLR for both filter states compared to the uncoated control filter can be ruled out, especially when considering that photon flux was even slightly higher for the absorptive filter state relative to the transmissive (0.25% log quanta/cm^2^/s). Also, differences in pupil response driven by chromatic pathways (due to different wavelengths) have been previously described as being 3-fold larger than those driven by luminance pathways (due to different intensities) [52]. However, photon flux reaching the retina also depends on the pupil area itself, which in turn is modulated by photon flux and the wavelength of the emitting light source [45]. This may be another factor to take into account when considering the obtained results in pupil size, and then regarding the potential filtering effect on light transmission to the central pacemaker (SCN). One limitation of this study is that a single illumination level and color temperature were tested. A wider illumination and color temperature range would allow to determine the irradiance-response relationship, not only regarding PLR, but also in terms of color perception, as well as to be able to generalize our conclusions to a wider lighting spectrum range implemented in home and work environments.

Another potential confounding factor could be the sleep-wake history, which can affect the light-dark exposure pattern, and in turn, the non-visual light responses [53]. In this case, we did not ask participants to maintain a stable sleep-wake pattern before the study, which may slightly influence the response.

To summarize, although at this stage we could not find significant differences between both states, there is an effect on the pupil response with the absorptive ECF compared to uncoated sample, which indicates a possible protection of circadian activation under blue light exposure. Additional studies with experimental melatonin evolution monitored over longer time periods, as well as behavioral and sleep studies involving chronodisruptive interventions, will allow to confirm and quantify these results in functional terms. Then, this novel filtering system could find application in several nocturnal excessive-irradiance sensitive scenarios: personal devices used by shift workers during nighttime to reduce blue light exposure (or during daytime to mitigate the effects of that night exposure). Another application of this ECF would be building-integrated devices in the form of tintable windows to prevent outside light pollution or to adjust light exposure during the first hours of the day in high latitude regions (especially in summer season), or as a filtering system for lighting sources installed in sensitive spaces, like hospitals, where patients can be submitted to excessive irradiance during nighttime control protocols. Although our color naming results are promising, this test may have inter-subject variability, so further studies on color perception using different approaches (e.g. matching techniques) could be helpful to confirm the usefulness of this system in contexts where instruments/materials are color coded, like in intensive care units, where a correct color identification relative to unfiltered standard light sources is essential.

Presenting the α-opic log photon irradiance as a function of wavelength for each photoreceptor’s maximum sensitivity provides an additional perspective of the processes going on during light exposure under the different filter states. The differences found are consistent with transmittance spectrum of each filter state. On the one hand, spectra of the control sample and the ECF in the transmissive state are similar, showing no absorption peak in the 400–700 nm range, and only differ in their transmittance values. Accordingly, activation values for the different photoreceptors show similar trends in both cases, even showing an almost constant difference of 0.2 log quanta/cm^-2^s^-1^, which can be attributed simply to the transmittance differences. This could be further enhanced by increasing transmittance of the transmissive state, so that reduction in short wavelength light exposure during daytime would be minimized. On the other hand, clear differences can be found for the filter in the absorptive state, especially for cyanopic and melanopic values, that is in the 400–500 nm range, corresponding to the range of the absorption peak of ECP-Yellow. The isosbestic point around 520 nm sets the frontier beyond which differences are less pronounced; this can be seen for the rhodopic values in particular, where both ECF states are very similar. Even for chloropic and erythropic values there is an inversion in the response of the two, accordingly to the higher transmittances shown for absorptive states in that region.

These results are closely related to theoretical melatonin suppression, that was assessed over a wide range of light sources and illuminances. The absorptive state perceptibly reduced theoretical melatonin suppression in all blue-containing light sources (similarly to previous experimental results on static filtering systems [54–57]). However, because of the limitations of this model in real life context [58] that have been discussed, it is possible that these particular results may be slightly overestimated. This model includes the blue-ON/yellow-OFF color opponency phenomenon, which is not known to be present in ipRGCs. Thus, future experiments on melatonin suppression under wider range of intensities and spectra will shed light on the real melatonin protection effects of this system. This will provide correlations between melatonin suppression and PLR parameters, which would show the relative contributions of different photoreceptors.

## Conclusions

Conceived as a proof of principle, in this study we applied dynamic blue light filtering by electrochromic materials to a physiological process in humans, such as pupil light reflex, anatomically related to circadian entrainment. Additional experiments involving protocols of circadian disturbing interventions (e.g. shift-work simulation) would be helpful to quantify the effectiveness of the electrochromic filter to prevent the circadian clock from disruption in altered light-dark cycle environments, as well as melatonin suppression. On the other hand, further studies related to visual phenomena beyond color perception will be desirable. With the limitations previously stated, we were able to confirm our ECF has certain ipRGC protecting effects (although no significant differences between both states were found) with limited changes in color naming tests. The limitations of the outcomes of this study have pointed out the possibility to further enhance this dynamic filtering system by increasing the transmissivity of the electrochromic polymer’s non-absorptive state as well as the transmissivity of the transparent conducting substrates. This will pave the way for integrating safe dynamic light filtering system using electrochromic devices in work environments where color is essential (e.g. hospitals). With the appropriate improvements, this system could help to regulate detrimental effects of inadequate light exposure in sleep and circadian disorders in environments where inadequate light exposure is mandatory (e.g. shift work).

## Supporting information

S1 File (DOCX)

S1 Data (ZIP)
