# Supplementary material for: Electrochromic selective filtering of chronodisruptive visible wavelengths
Source: PLoS One. 2020 Nov 5;15(11):e0241900. doi: 10.1371/journal.pone.0241900 (PMC7643985; doi:10.1371/journal.pone.0241900)
Supplement: S1 File — (DOCX) [file pone.0241900.s001.docx]

**SUPPORTING INFORMATION**

**Synthesis of ECP-Yellow**

In short, ECP-Yellow was prepared by direct arylation polymerization of the dibromo derivative of 3,4-bis((2-ethylhexyl)oxy)thiophene-co-2,5-dimethyloxybenzene and the dihydro analog, both functionalized with 2-ethylhexyloxy side chains (R in Scheme 1). Palladium acetate Pd(OAc)_2_ was used as the catalyst, pivalic acid and K_2_CO_3_ as the proton shuttle and base, respectively. Dimethylacetamide (DMAc) was used as the solvent. The polymers were purified via Soxhlet extraction using methanol, acetone, hexanes, and finally chloroform. Impurities were further removed using palladium and potassium scavengers. The molecular weight determined by gel permeation chromatography to was M_n_ = 10.9 kDa, M_w_ = 22.5 kDa, Đ = 2.1 versus polystyrene standards. The purity was confirmed by ^1^H NMR, ^13^C NMR, and elemental analysis [1] .

| 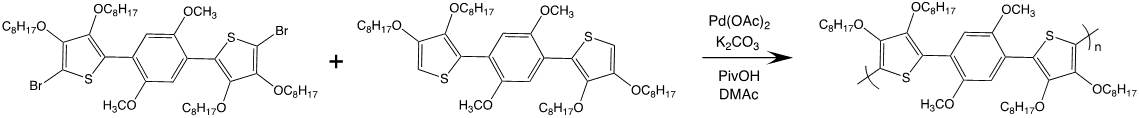 |
| --- |
| **Scheme 1**. Synthesis route of ECP-Yellow |

**Materials optimization and filtering properties**

*Materials and fabrication*

Lithium trifluoromethanesulfonate (LiCF_3_SO_3_) (96 %, Aldrich), propylene carbonate (PC) (99%, Aldrich), vanadium (V) triisopropoxide oxide (96 %, Alfa-Aesar), 2-propanol (ACS, 99.5%, Alfa-Aesar), toluene (99.9 %, Aldrich), poly(ethyleneglycol)diacrylate (Aldrich), 2,2-dimethoxy-2-phenylacetophenone (99 %, Aldrich), and glass beads (< 106 μm, Aldrich), were used as received. ITO glass (4-8 ohms/sq, Delta Technologies) and ITO-PET (60 ohms/sq, Aldrich) were used as substrates.

*Equipment.*

An airbrush spray gun (Iwata-Eclipse HP-BC) connected to a nitrogen line, mounted on a CNC station (HEIZ, CNC High-Z S-series) was used for deposition of ECP-Yellow and V_2_O_5_ films. A Cary 50 UV-Vis spectrophotometer and an Ocean-Optics 2000 spectroradiometer were used for optical characterization of materials and filters. A Biologic SP-50 potentiostat was used for the electrochemical measurements and for driving the electrochromic filter (ECF). A UV-lamp (Vilber-Loumat VL-4-L, 365 nm, 4W) was used to cure the electrolyte gel.

*Characterization*

ECP-Yellow shows a color transition between a highly transparent oxidized state and a vibrant yellow in its neutral state, with an absorbing region in the 400-500 nm (with λ_max_ at 446 nm and a flat, non-absorbing region in 500-700 nm (Fig S1a). Optical characteristics of a thick enough film of ECP-Yellow match with those of a long-pass filter with a cut-on frequency of approx. 500 nm (Fig S2). “Conventional” filtering of increasing thickness films was assessed under different commercially available light sources, confirming filtering properties below 500 nm, together with negligible effects for higher wavelengths in the eye sensitivity range (400-700 nm) (Fig S2).

| 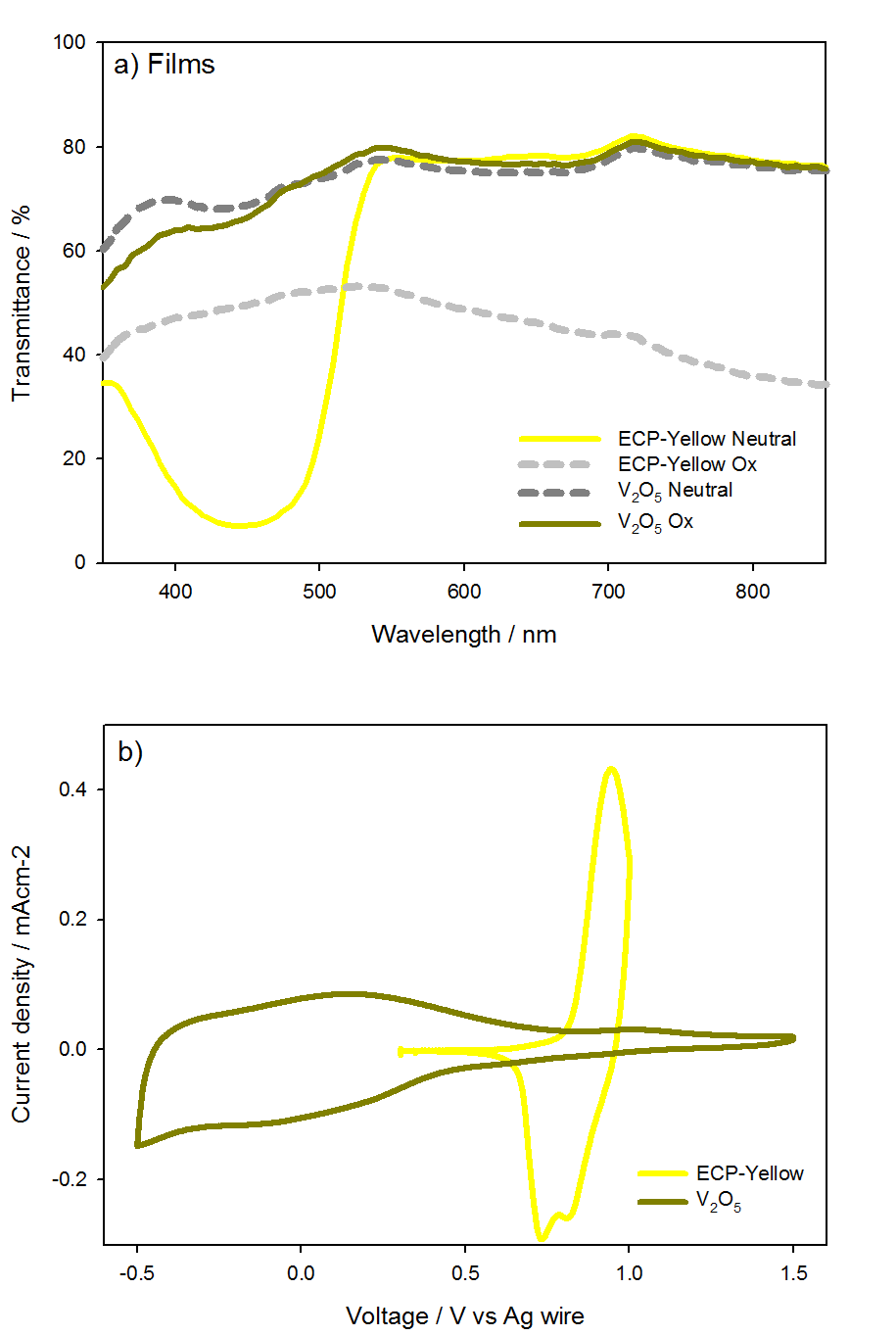 |
| --- |
| **Figure S1.** Transmittance spectra of a) ECP-Yellow and V_2_O_5_, in their neutral and oxidized states (transmittance of ITO substrate included). b) Characteristic cyclic voltammograms of ECP-Yellow and V_2_O_5_ films, conducted at 20 mV/s in a 0.1 M LiClO_4_ (PC) solution. |
| 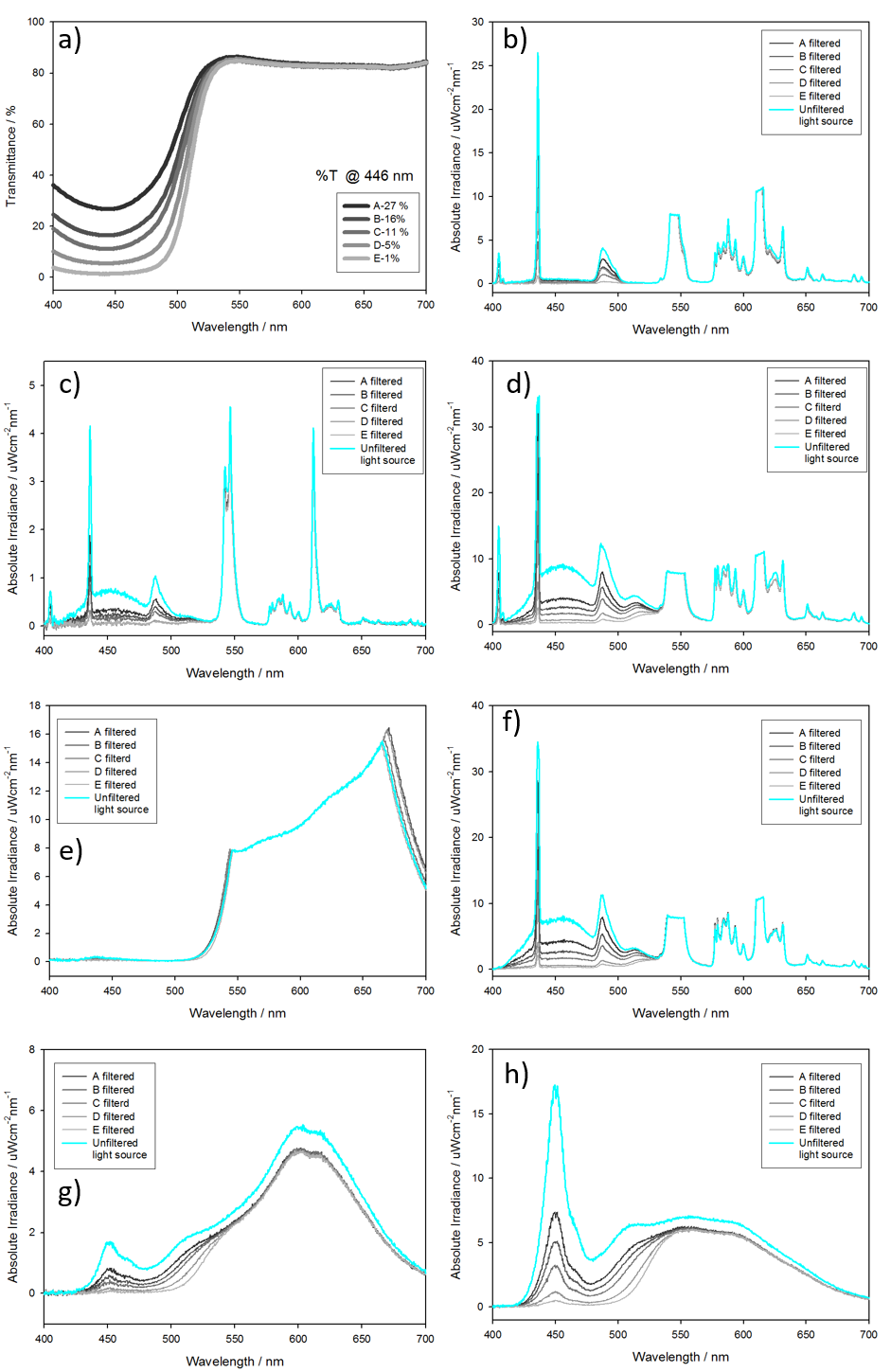 |
| **Figure S2.** Static filtering of ECP-Yellow films.a) Transmittance spectra of five different ECP-Yellow films of increasing thickness. The corresponding spectra are labelled as A-E, corresponding with the following transmittance values at 446 nm: A: 27 %, B: 16 %, C: 11 %, D: 5 % and E: 1 %.  (b-h) The rest of figures represent the increasing levels of filtering achieved with those films for different commercially available light sources. Light blue lines in each figure represent the absolute irradiance spectra of the corresponding light source, while gray-scaled lines represent the spectra filtered by each of the films (A-E) characterized in figure a. |

Electrochromic properties of this material, however, can add a substantial difference to conventional filters, with the ability of dynamically change its absorbing spectra. To maximize the dynamic effect, differences in transmittance spectra (contrast) should be optimized. To define the maximum contrast obtainable and the corresponding deposition conditions, an optimization study of ECP-Yellow was carried out following the procedure described elsewhere [2,3]. According to it, a number of films with increasing thickness/redox capacity were optically and electrochemically characterized. Electrochemical characterization was performed via cyclic voltammetry (Fig. S1b), where applied potential was repeatedly swept between a previously defined potential window, and the charge related to the electrochromic process, i.e. the redox capacity of the material, can be obtained through the proper integration of the I-V curve. Transmittance spectra in both transmissive and absorptive states were obtained (Fig. S1a) for the optical characterization. Linear relationships between absorbance and redox capacity for both transmissive and absorptive states were found (Fig. S3a). Translating these values to transmittance values two exponential decays were obtained, which subtraction represents the contrast of the corresponding films, this curve showing a maximum value. This contrast value was identified as 58 ± 5 % (in agreement with previously reported values [1]) and obtainable with a film of 3.1 ± 0.8 mCcm^-2^ redox capacity.

| 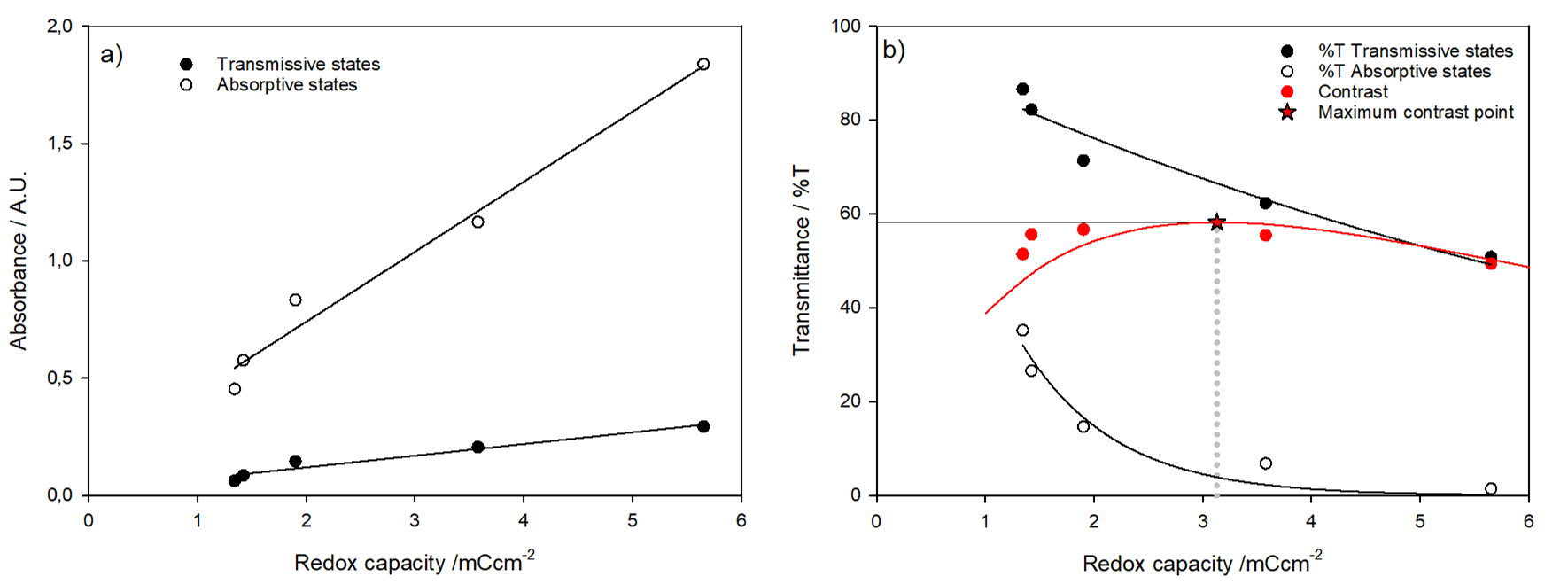 |
| --- |
| **Figure S3.** Optimization procedure for ECP-Yellow films. (a) Linear evolution of absorbance in transmissive and absorptive states as a function of redox capacity, for sprayed ECP-Yellow films. (b) Corresponding exponential decays of the transmittance values, and contrast plot for the same films. The red star (at the intersection of the dotted lines) identifies the maximum contrast value. |

To develop functional devices, V_2_O_5_ was chosen as an appropriate complementary material. Vanadium pentoxide has been reported to show negligible color transitions under electrochemical switching for thin enough films, therefore being a candidate to act as transparent electroactive counter electrode material. Films of approximately 3 mCcm^-2^, were able to compensate the redox capacity necessary to completely switch the optimized ECP-Yellow films, showing minimum contributions to the transmittance spectra (Fig. S1a). To characterize and optimize the ECP-Yellow and the V_2_O_5_ films, optical and electrochemical measurements were carried out in a cuvette sized glass cell (12 mm×12 mm×45 mm); a silver wire was used as a pseudo-reference electrode (calibrated 0.1 V vs. NHE), a stainless steel plate as the counter electrode, and 0.1 M LiCF_3_SO_3_-propylene carbonate (PC) as the electrolyte.

**Device fabrication and characterization**

Electrochromic filters with a 5x5 cm^2^ active area were fabricated with a “sandwich-type” architecture (Figs. S4a and S4b). ECP-Yellow films were deposited on ITO substrates from a solution of 5 mg/ml polymer in toluene. V_2_O_5_ films were deposited according to the procedure based on the one proposed by Hazjeri M. *et al*. [4] (same conditions except the initial precursor solution concentration, which in this case was 0.025 M vanadium triisopropoxide in isopropanol). Films of each material were spray coated via an automated CNC routine, assuring reproducibility of each deposition. Gas pressure and gun-substrate distance were kept fixed during all the experiments (0.5 bar, 6 cm). Optimized films for devices (deposited over a surface area of 5 x 7.5 cm^2^, with a final active surface area of 5x5 cm^2^) were obtained with 380 μl (corresponding to approx. 15 μl/cm^2^) for ECP-Yellow and 125 μl (corresponding to 5 μl/cm^2^) for V_2_O_5_ films, respectively. Devices were assembled using a photo-crosslinkable electrolyte gel [5], exposed to UV-light for 10 minutes.

| **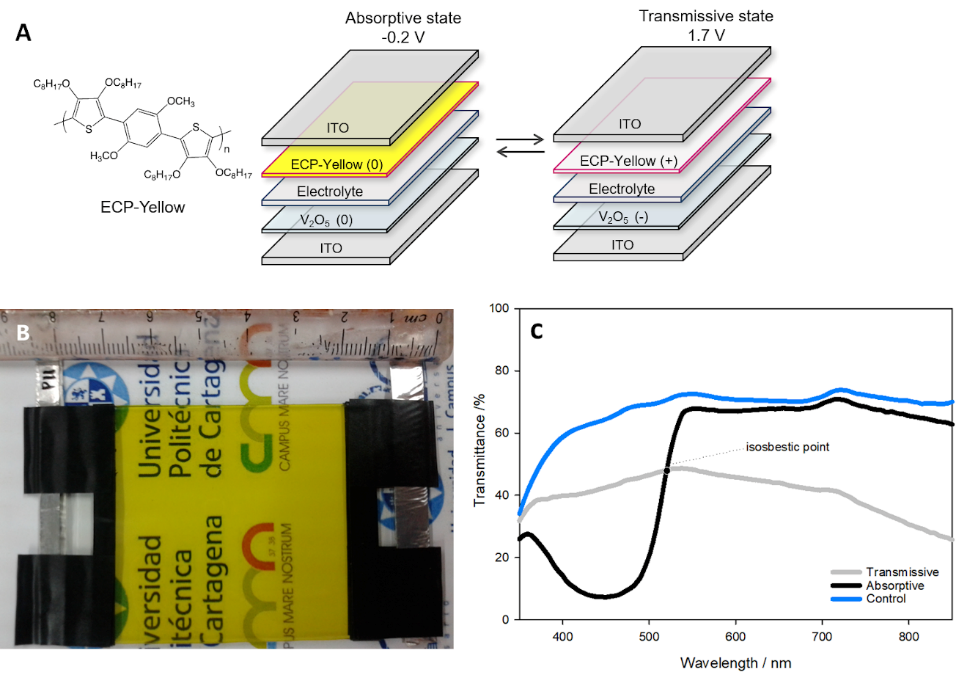** |
| --- |
| **Figure S4.** a) “Sandwich-type” architecture of the electrochromic devices b) Picture of a 5x5 cm^2^ active area device as fabricated c) Transmittance spectra of control device (blue line), and transmissive (grey line) and absorptive (black line) states for the rest of devices. |

Transmissive and absorptive states of the ECFs were obtained by applying 1.7 V and -0.2 V, respectively. A reproducible contrast (difference of transmittance between the two states measured at 446 nm) of 32 ± 3 % was obtained for all devices. These values correspond to the complete device transmittance, including electrochromic materials, substrates, and electrolyte gel. To determine the influence of the electrochromic material itself apart from the remaining device components, a control sample composed of just uncoated substrates and gel was also assembled. Photographs of the ECF are shown in Figure S4b, while characteristic spectra of the ECF in its transmissive and absorptive states along with that of the control sample are shown in Figure S4c. As a preliminary assessment of our fabrication process and performance of the resulting devices, we tested them under two different conditions: First, to assess reproducibility of the fabrication process and optical response, freshly prepared devices were used for some of the participants (11 out of 16); second, to assess reproducibility in multiple switching conditions, the rest used the same device repeatedly switched. With the first set of devices we were able to assess reproducibility in our fabrication processes (showing consistent contrast values) while the second set confirmed the ability of the devices to undergo repeated switches without loss in electrochromic dynamic filtering.

Table S1. Munsell cards properties

| Munsell ref. | # | RGB Hexdec | RGB 0÷255 | RGB % | CMYK % | HSV | HSL | CIE-L*ab | XYZ |
| --- | --- | --- | --- | --- | --- | --- | --- | --- | --- |
| 10BG 6/4 | 1 | 699DAA | 105, 157, 170 | 41.2, 61.6, 66.7 | 38, 8, 0, 33 | 192°, 38, 67 | 192°, 28, 54 | 61.7, -13.8, -12.2 | 25.1, 30, 42.5 |
| 5Y 6/4 | 2 | A79367 | 167, 147, 103 | 65.5, 57.6, 40.4 | 0, 12, 38, 35 | 41°, 38, 65 | 41°, 27, 53 | 61.7, 1, 26 | 28.8, 30.1, 17.1 |
| 7.5R 6/4 | 3 | BA8989 | 186, 137, 137 | 72.9, 53.7, 53.7 | 0, 26, 26, 27 | 0°, 26, 73 | 0°, 26, 63 | 61.8, 18.7, 7.4 | 33.7, 30.1, 27.7 |
| 2.5G 6/6 | 4 | 65A27C | 101, 162, 124 | 39.6, 63.5, 48.6 | 38, 0, 23, 36 | 143°, 38, 64 | 143°, 25, 52 | 61.7, -28.3, 13.6 | 21.9, 30.1, 23.7 |
| 5GY 6/8 | 5 | 899E38 | 137, 158, 56 | 53.7, 62, 22 | 13, 0, 65, 38 | 72°, 65, 62 | 72°, 48, 42 | 61.7, -22.2, 49.1 | 23.3, 30.1, 8.3 |
| 10P 6/8 | 6 | C080B7 | 192, 128, 183 | 75.3, 50.2, 71.8 | 0, 33, 5, 25 | 308°, 33, 75 | 308°, 34, 63 | 61.7, 33.4, -18.9 | 38, 30.1, 48.6 |
| 2.5P 6/8 | 7 | A389CD | 163, 137, 205 | 63.9, 53.7, 80.4 | 20, 33, 0, 20 | 263°, 33, 80 | 263°, 40, 67 | 61.7, 23.6, -31.5 | 35.1, 30.1, 61.7 |
| 5PB 6/8 | 8 | 7495D5 | 116, 149, 213 | 45.5, 58.4, 83.5 | 46, 30, 0, 16 | 220°, 46, 84 | 220°, 54, 65 | 61.7, 5.5, -36.3 | 30, 30, 67.2 |

Munsell cards properties: RGB (red-green-blue) Hexadecimal code (RGB Hexdec); decimal color code (RGB 0÷255); RGB (R%, G%, B%) percent representation of color; CMYK (Cyan-Magenta-Yellow-Black) (C%, M%, Y%, K%) representation of color; hue, saturation and value (HSV) and hue, saturation, and lightness (HSL) representation of color; CIELAB color space (CIE-L*ab, L* = lightness from black (0) to white (100), a* from green (−) to red (+), and b* from blue (−) to yellow (+)); CIE XYZ coordinates.


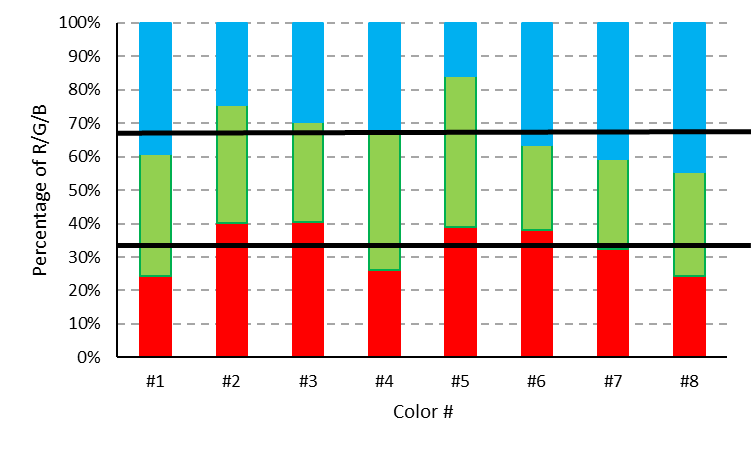


**Figure S5. Red/Green/Blue (**R/G/B) content for each color selected. Black solid horizontal lines are just indicative of 33-67 %. Colors with more than 33 % of blue are expected to show lower hit rate in color naming tests under absorptive states. Colors with more than 67 % of red/green are expected to show lower hit rate in color naming tests under transmissive state.

**Figure S6.** **Normalized spectra for unfiltered light sources used for theoretical melatonin suppression.**

**Results**

**Color Naming**

**
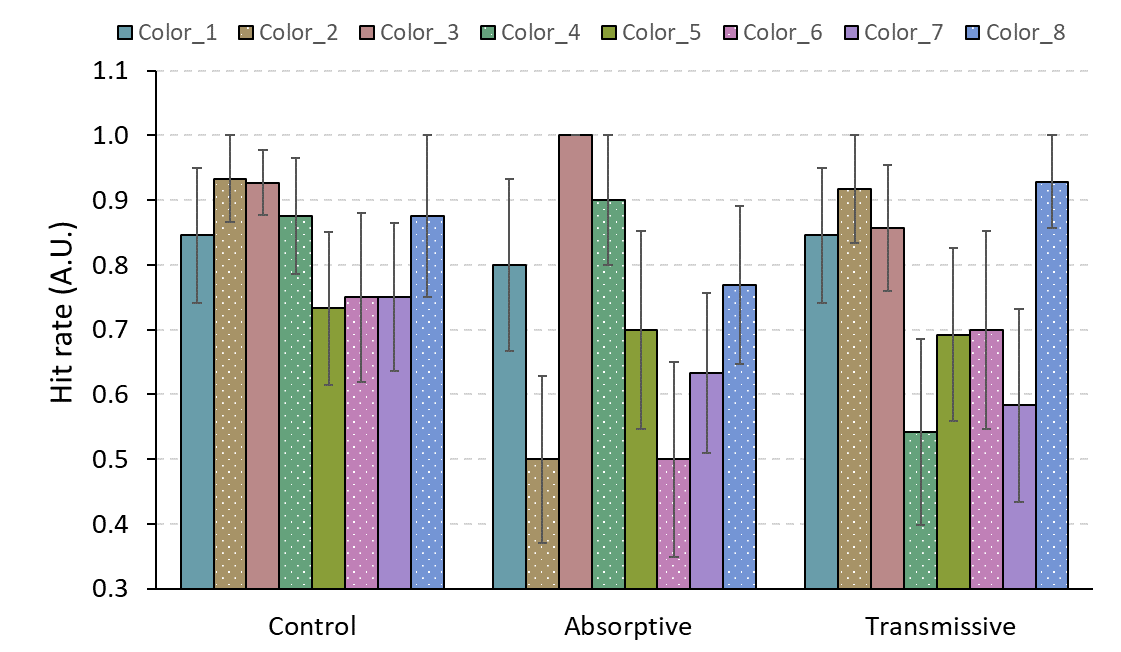
**

*

**Figure S7.** Hit rate in naming for each color shown under each ECF state. * indicates significant differences (p < 0.05) compared with control.

**Suppl. References**

[1] K. Cao, D.E. Shen, A.M. Österholm, J.A. Kerszulis, J.R. Reynolds, Tuning color, contrast, and redox stability in high gap cathodically coloring electrochromic polymers, Macromolecules. 49 (2016) 8498–8507. doi:10.1021/acs.macromol.6b01763.

[2] J. Padilla, V. Seshadri, G.A. Sotzing, T.F. Otero, Maximum contrast from an electrochromic material, (2007). doi:10.1016/j.elecom.2007.05.004.

[3] J. Padilla, A.M. Österholm, A.L. Dyer, J.R. Reynolds, Process controlled performance for soluble electrochromic polymers, Sol. Energy Mater. Sol. Cells. 140 (2015) 54–60. doi:10.1016/j.solmat.2015.03.018.

[4] M. Hajzeri, A. Šurca Vuk, L. Slemenik Perše, M. Čolović, B. Herbig, U. Posset, M. Kržmanc, B. Orel, Sol–gel vanadium oxide thin films for a flexible electronically conductive polymeric substrate, Sol. Energy Mater. Sol. Cells. 99 (2012) 62–72. doi:10.1016/J.SOLMAT.2011.03.041.

[5] V. Seshadri, J. Padilla, H. Bircan, B. Radmard, R. Draper, M. Wood, T.F. Otero, G.A. Sotzing, Optimization, preparation, and electrical short evaluation for 30 cm^2^ active area dual conjugated polymer electrochromic windows, Org. Electron. 8 (2007) 367–381. doi:10.1016/J.ORGEL.2007.01.004.

**ANNEX 1 - INFORMED CONSENT FORM. APPROVED BY THE UNIVERSITY OF MURCIA ETHICS COMMISSION. REFERENCE ID 2000/2018.**

D./Dña ……………………………………………………………………………, de ….. años de edad y con DNI nº ………..….., manifiesta que ha sido informado/a sobre los beneficios que podría suponer mi participación para cubrir los objetivos del Proyecto de Investigación titulado “NUEVOS DESARROLLOS TECNOLÓGICOS PARA LA EVALUACIÓN Y PREVENCIÓN DE LA CRONODISRUPCIÓN EN POBLACIÓN SENSIBLE”, dirigido por Dª. María de los Ángeles Bonmatí Carrión.

Dicho proyecto se llevará a cabo en el LAIB y/o la Facultad de Biología y cuenta con el certificado del Comité Ético correspondiente. El teléfono de contacto de la investigadora responsable es: 868884937 y correo electrónico: [mabonmati@um.es](mailto:mabonmati@um.es). Este proyecto está financiado por la Fundación Séneca, con el fin de determinar la relación entre el sueño, la disrupción circadiana y los estilos de vida, considerando los principales sincronizadores circadianos (luz, ejercicio y alimentación) en individuos propensos a la cronodisrupción

He sido informado/a de los posibles perjuicios que la participación en dicho proyecto puede tener sobre mi bienestar y salud al haber leído la hoja de información al participante sobre el estudio citado. **(el resto de información se daría en el documento hoja informativa)**

He sido también informado/a de que mis datos personales serán sometidos a tratamiento en virtud de su consentimiento con fines de investigación científica por la Universidad de Murcia. El plazo de conservación de los datos será el mínimo indispensable para asegurar la realización del estudio o proyecto. No obstante, mis datos identificativos, para garantizar condiciones óptimas de privacidad, y cuando el procedimiento del estudio lo permita, podrían ser sometidos a anonimización o seudoanonimización. En todo caso, la información identificativa que se pudiese recabar será eliminada cuando no sea necesaria.

He sido informado/a de que para cualquier consulta relativa al tratamiento de sus datos personales en este estudio o para solicitar el acceso, rectificación, supresión, limitación u oposición al tratamiento podré dirigirme a la dirección protecciondedatos@um.es. Asimismo he sido informado/a de mi derecho a presentar un reclamación ante la Agencia Española de Protección de Datos.

He sido también informado de que puedo abandonar en cualquier momento mi participación en el estudio sin dar explicaciones y sin que ello me suponga perjuicio alguno.

Se me ha entregado una hoja de información al participante y una copia de este consentimiento informado, fechado y firmado.

Tomando ello en consideración, **otorgo** mi **consentimiento** a que esta recogida de datos tenga lugar y sea utilizada para cubrir los objetivos especificados en el proyecto.

, a de de 20 .

Fdo. D/Dña
